# Comparison of Resilience, Positive/Negative Affect, and Psychological Vulnerability Between Iranian Infertile and Fertile Men

**Published:** 2013

**Authors:** Abbas Abolghasemi, Saied Rajabi, Moslem Sheikhi, Azar Kiamarsi, Vida Sadrolmamaleki

**Affiliations:** 1 Department of Psychology, University of Mohaghegh Ardabili, P.O. Box: 179, Ardabil, Iran.; 2 Ardebil Branch, Islamic Azad University, Ardebil, Iran.

**Keywords:** Infertile, Resilience, Positive/Negative Affects, Psychological Vulnerability

## Abstract

**Objective**: To compare resilience, positive/negative effect, and psychological vulnerability between fertile and infertile men.

**Methods:** The research sample consisted of 40 fertile and 40 infertile men who were selected among men who presented to an infertility clinic. To collect data, Connor–Davidson Resilience Scale, Positive/Negative Affect Schedule, and Brief Symptoms Inventory were used.

**Results: **The MANOVA results showed that infertile men had higher mean (SD) score for negative affect (46.15±8.31 vs. 23.10±8.50) and psychological vulnerability (37.90±12.39 vs. 23.30±6.40) than fertile men (P= 0.001); while infertile men had lower resilience (59.35±14.25 vs. 82.17±13.03) and positive affect (43.01±10.46 vs. 61.85±8.14) than fertile men (P= 0.001).The results of multiple regressions showed that resilience and negative affect had the highest significant contribution in prediction of psychological vulnerability in the infertile.

**Conclusion: **Resilience and negative effects are the best predicators for mental vulnerability of infertile men. These factors may be addressed in future studies in infertile men.

**Declaration of Interest:** None.

## Introduction

Infertility is one of the crucial crises in human life which may lead to mental problems and experience of vulnerability in affected individuals (1). Infertility experience has been associated with social, psychological and physical stresses affecting all aspects of life (2). There is continued debate related to the most accurate conceptualization of psychological features associated with infertility problems (3). Based on the empirical evidence, it appears that the stress of infertility is related to increased rates of depression and anxiety (4).Domtar et al. (5) found that the rate of anxiety and depression in infertile women equals with the rate of anxiety and depression in those with heart attack, cancer and AIDS. 

However, the vast majority of individuals coping with infertility problems do not exceed clinical thresholds for these disorders. In a large study of 2,250 individuals dealing with infertility, two thirds of women and nearly three-fifths of men agreed that infertility strengthened their relationship and brought them closer together (6). Consistent with bio-psychosocial models of infertility, the reactions to infertility problems may be best characterized as contextually determined by the interplay between interpersonal relationships, physiological parameters, risk and protective factors, cultural expectations, and individual coping resources (7).

For those dealing with infertility issues, in addition to the stressors related to the experience of involuntary childlessness itself, medical treatment is often additionally quite taxing. Care may involve multiple and invasive treatment cycles that are commonly unsuccessful, economically and personally draining, and generally without a clear endpoint in the absence of parenthood. Psychological factors and difficulties coping with the emotional demands of treatment have been implicated in the high rates of treatment dropout (8). In Iran, Bhatia Adriana et al. (9) showed that the infertile women (5.08%) have greater psychological vulnerability regarding mental health than infertile men (2.3%).

Resilience has yet to be examined in the context of infertility, despite the known relationships between resilience and other medical conditions. Resilience has been broadly defined as the capacity of individuals exposed to a negative event to ‘‘maintain relatively stable, healthy levels of psychological and physical functioning” (10) and to ‘‘cope flexibly with life’s challenges” (11). Despite the myriad definitions used to operationalize this construct, resilience appears to serve as a protective factor to reduce the impact of stressors. Moreover, resilient persons are better prepared to use active and social coping methods (12, 13).

More recently, healthcare scientists have begun to appreciate the possible associations between resilience and psychological and physical illness as well as the importance of understanding wellbeing, positive functioning, and self-actualization (14). Banana and Loss (10) stated that resilience facilitates the preservation of functioning and is a marker of wellbeing rather than simply the ‘‘absence of pathology”. Despite this distinction, resilience appears to decrease the propensity to experience depression and anxiety (15). Similarly, characteristics of resilience have been associated with decreased disease susceptibility, improved prognosis, and better adaptation to chronic conditions such as cancer (16), HIV (17), cardiac disease (18), arthritic pain (19), and diabetes (20).

Peterson et al. (21) investigated the relationships between specific coping strategies, infertility-specific stress, and depression. They observed that the social support seeking and problem solving coping were negatively related to infertility-specific distress and depression while the reverse was demonstrated for escape-avoidance, accepting responsibility, and distancing strategies. Sexton et al. (7) showed that resilience was negatively associated with infertility-specific and general distress. Engagement in action-focused coping skills was positively correlated with resilience. Unlike coping, resilience has yet to be examined in the context of infertility, despite the known relationships between resilience and other medical conditions. 

Spinoza (22) proposed that all emotions can be derived from the basic emotions pleasure and pain. Today, a different distinction between emotional states is advocated, namely, positive affect versus negative affect (23). Negative affect includes symptoms of anxiety and depression, whereas cheerfulness and joy comprise examples of positive affect (24). Contrary to common misconception, positive affect and negative affect can co-occur at the same time within an individual (25), indicating that they do not merely exist at opposite ends of a continuum (26).

Despite the relative independence of positive affect and negative affect (25), this has not translated into a balanced investigation of their impact on health outcomes, as research has largely focused on negative effect, thereby neglecting the role of positive emotions (24).

However, accumulating evidence suggests that positive affect may be important in enhancing our understanding of health (27). In fact, it may be more valuable to study negative affect and positive emotions in concert as predictors and modulators of health outcomes, as these mood states may interact (24).

Most infertile individuals consider the evaluation and treatment of infertility to be the most upsetting experience of their lives (28). In a review, Greil (4) reported that majority of studies have shown that infertile couples differ moderately from fertile norms on at least some indices, especially those related to interpersonal sensitivity and depression. In addition, psychological distress appears to be more common in the partner with the fertility problem (29).

In view of the above, the present study attempted to extend or improve upon the previous research in one way. The research that examines the comparison of resilience, positive/negative affect and psychological vulnerability of infertile and fertile men could be useful. In this research, resilience, positive effect, negative affect, and mental vulnerability as dependent variables are compared between fertile and infertile men.

## Materials and Methods

*Participants*

The research sample consisted of 40 infertile men who referred to the infertility center of Mahdieh Hospital of Tehran, Iran in 2010. They were selected through convenience sampling method. Then, 40 men who had the experience of fertility were selected as normal individuals for the study. The required data were gathered via questionnaires in the following order: Brief Symptoms Inventory (BSI), Connor–Davidson Resilience Scale (CD-RISC), and Positive and Negative Affect Schedule (PANAS).

Inclusion criteria were age range of 30-40 years, minimum education level of high school diploma, being employed, married for at least 5 years, and no history of chronic physical and/or mental disease.

Then subjects were asked to carefully complete the inventories (see below). After collecting all data, it was statistically analyzed using statistic methods of multivariate analysis of variance (MANOVA) and multivariate regression analysis.

***Measures***

Instruments utilized for data collection were as follows:

*Connor–Davidson Resilience Scale (CD-RISC) *(15) :

This is a 25-item scale that measures the ability to cope with stress and adversity. Responses are indicated on a 5-point Likert scale ranging from 0 to 4 (score range, 0–100 for scale). Internal consistency of the CD-RISC has been reported as 0.87 (30).

*Positive and negative affect schedule (PANAS)* (31):

This schedule consists of 10 negative and 10 positive mood terms. Responses are indicated on a 7-point Likert scale ranging from 0 to 4 (score range: 0–70 for each subscale). The validity and internal consistency of the positive (α= 0.88) and negative affect (α=.87) schedule are good, with the test–retest reliability being the highest for the “general” temporal instruction (31). In a study (32), internal consistency of the scales was satisfactory; higher values for recalled poor performance (PA scale α= 0.83, NA scale α= 0.84) compared to recalled optimal performance (PA scale α=0.79, NA scale α= 0.73) were found. Bakhshipoor and DejhKam (33) reported the internal consistency for this schedule as 0.87. Also, the positive affect scale(r= 0.52), and negative affect scale (r= -0.43) were correlated with Psychological Well-being Scale (P< 0.01) (33).

*Brief Symptoms Inventory (BSI-18):*

This is a self-report measure consisting of 18 items (34). Participants indicate their response on a five-point scale. Responses are indicated on a 5-point Likert scale ranging from 0 to 4 (score range: 0–72 for the inventory). The general index obtained a Cronbach’s α of 0.89, and the Cronbach’s α of three classic subscales of somatization, depression, and anxiety were 0.80, 0.86, and 0.73, respectively. However, when the anxiety dimension was considered as two subscales (empirical structure), the indexes obtained were lower than 0.70 (0.67 for the panic factor and 0.65 for the general anxiety factor). Modanlo (35) reported the internal consistency for the inventory as 0.85. He also showed that the positive affect scale (r= -0.66), and negative affect scale (r= 0.41) were correlated with BSI (p< 0.01).

The gathered data were analyzed using multiple analysis of variance (MANOVA) and multiple regression analyses using the SPSS software for Windows (ver. 18.0).

## Results

Mean (±standard deviation, SD) age of infertile men was 35.07 (±7.2) years, and that of fertile men was 34.58 (±7) years. Nineteen men were within the age range of 30-40 years. Also, the mean of their literacy was 13.32.

Table 1 shows means (ISD) and MANOVA of variables used in the analyses. The MANOVA results showed that there were significant differences regarding resilience (F=63.15), positive affect (F=39.41), negative affect (F=50.40) and mental vulnerability (F=43.81) between fertile and infertile men (p<0.01). The results showed that infertile men had lower resilience, lower positive affect, higher negative affect, and higher mental vulnerability than fertile men.

As seen in table 2, MANOVA showed that there were significant differences between fertile and infertile men regarding resilience, positive, negative affect, and mental vulnerability. 

The root square of Eta shows that the difference between two groups is significant with regard to dependent variables estimated at 74% (i.e., 74% of variance is related to the difference between two groups due to the effects of dependent variables).

To determine the impact of each variable (i.e., resilience, positive and negative affect) as predictive variables and mental vulnerability as criterion variable were analyzed in regression equation. Table 3 shows that F value is significant and 61% of the variance of mental vulnerability is explained by variables of resilience, positive and negative affect (adjusted R2=0.611, F=36.76, p<0.001). Considering Beta values, resilience (β=–0.739), negative affect (β=0.424) and positive affect (β=-0.333) can predict significantly the changes relevant to mental vulnerability in infertile men.

The results of t-test showed that the value of coefficient and β regression of variable are significant and the variables of resilience, positive and negative affect have significantly affected the mental vulnerability in infertile men.

## Discussion

The purposes of the study were to compare the resilience and negative/positive affect between fertile and infertile men, and to determine their roles in predicting the mental vulnerability.

The results of the study showed that the mean of resilience in infertile men is significantly less than that of fertile men (p<0.01). The indication of vulnerability in infertile men proves that invulnerability as a source of inner resilience can lessen the negative affect of stress, preventing physical and mental disorders. It filters down human defense against stress in dealing with problems (36).

**Table 1:** Means, standard deviations, and MANOVA of resilience, positive/ negative affect and mental vulnerability in infertile and fertile men

	**Fertile**	**Infertile**					
	**Mean (±SD)**	**Mean (±SD)**	**SS**	**df**	**MS**	**F**	**Sig.**
**Resilience**	82.17±13.03	59.35±14.25	31549.61	1	31549.61	63.15	.0001
**Positive affect**	61.85±8.14	43.01±10.46	16646.45	1	16646.45	39.41	.0001
**Negative affect**	23.10±8.50	46.15±8.31	10626.05	1	10626.05	50.40	.0001
**Mental vulnerability**	23.30±6.40	37.90±12.39	04263.20	1	04263.20	43.81	.0001

**Table 2:** Multivariate tests on scores of resilience, positive/negative affect and mental vulnerability in infertile and fertile men

**Effect**	**Value**	**F**	**Sig**	**Partial Eta Squared**
**Pillai,s Trace**	0.742	53.82	.0001	.742
**Wilks Lambda**	0.258	53.82	.0001	.742
**Hotelling,s Trace**	2.870	53.82	.0001	.742
**Rots Largest Root**	2.870	53.82	.0001	.742

**Table 3:** Multiple regressions for predictor of mental vulnerability (i.e., resilience, positive and negative affect) in infertile men

**Predictors**	**MC** †	**RS** ‡	**ARS** §	**F(sig) ** ||	**b** ¶	**SE**	***B*** ††	**t(sig)** ‡‡
**R**	0.739	0.546	0.540	93.65(<0.001)	–.421	0.044	–.739	–3.03(<0.004)
**PA**	0.753	0.568	0.556	53.52(<0.001)	–.237	0.092	–.333	–1.11(0.27)
**NA**	0.782	0.611	0.595	39.76(<0.001)	0.363	0.125	.424	3.51(<0.001)

† Multiple Coefficient

‡ R Square

§ Adjusted R Square

|| F= F Test

¶ b Coefficient Standard Error,

†† Beta Coefficient

‡‡ t=T Test.

To cope with the various problems, the men of low resilience turn to proactive approaches by which they substitute risk-taking experience with mental pressure causing more anxiety or worries and restrains in unexpected disasters (37).

It is believed that the infertile men, evaluating the disastrous situations as more challenging rather than risk-taking and feeling more devotion to their duties, try to control all aspects of their lives and consider the stressors as an opportunity for transformation, making a contribution to preserve their mental safety. Since the infertile individuals are pessimistic, usually unable to handle with the problems, taking emotional approaches to difficulties, having negative attitudes toward the consequences (outcomes) of an action and considering them unrelated to each other, they reinforce their problems particularly in unexpected disasters (38).

The obtained results show a strong correlation with the findings of other studies (38-40).Tugged & Fredrichson (41) believed that low resilience would contribute to overcome the undesirable experiences by means of negative affects. Generally speaking, resilience might enhence one's endurance against difficulties creating positive attitudes toward life and fulfilling the requirements needed for satisfaction. Wolff (42) discussing the positive effects of resilience on mental safety, put an emphasis on such characteristics as social ability, creativity and capability in solving problems, self-efficacy, sense of purposefulness and better life expectancies.

The current study indicated that the infertile men experience negative emotions such as anger, hostility, depression, sadness, shy and grieve more than fertile men. On the other hand, the experience of positive emotions such as pride, happiness, joy, pleasure and confidence were less than fertile ones. These backgrounds provide a proper bed for creating mental problems and disorders. In other words, such experiences increase the vulnerability of infertile men. 

The results also showed that resilience and negative effects were the best predicators for mental vulnerability of infertile men. These results indicate that 61% of mental vulnerability variance can be accounted for by its variables. The findings have important implications for psychiatric interventions.

To elaborate the results of the study, it is stated that low resilience and negative emotions make the individual unable to encounter the tensions and their negative consequences (43). To justify the impact of negative emotions on social vulnerability, it would be reasonable to claim that negative affects result in increasing evaluation of risk-taking situations and decreasing individual expectation of his achievement (41).

Men of low resilience cannot overcome the negative affects and retain their mental safety; therefore, they face less anxiety and depression as the result of low resilience and negative affects (44). Considering the findings of previous research and the results of the present study, it is possible to enhance the resilience and facing with negative emotions by suggesting new psychological modalities in infertile men. Sternberg and Bery (45) believed that mental safety of infertile le men can be increased by teaching communicative skills, assertive triaging, etc.

The limitations of the study include the limited number of the subjects and this fact the samples were studied at an infertility clinic which may reduce the validity of the tests. It is suggested that enough attention should be paid to resilience to encounter the problems to reduce anxiety and tension and to use structural equation modeling. Also, the results have important implications for prevention of psychological problems in infertile men.

## Authors' Contributions

AA conceived and designed the evaluation and helped to draft the manuscript. SR participated in designing the evaluation and performed parts of the statistical analysis. Msh collected the clinical data. AK and VS revised the manuscript and performed the statistical analysis and revised the manuscript. All authors read and approved the final manuscript.
